# Perspectives and Challenges in Microbial Communities Metabolic Modeling

**DOI:** 10.3389/fgene.2017.00088

**Published:** 2017-06-21

**Authors:** Emanuele Bosi, Giovanni Bacci, Alessio Mengoni, Marco Fondi

**Affiliations:** Department of Biology, University of FlorenceFlorence, Italy

**Keywords:** microbial communities, metabolic modeling, constraint-based modeling, metabolic interactions, microbiome, mcFBA

## Abstract

Bacteria have evolved to efficiently interact each other, forming complex entities known as microbial communities. These “super-organisms” play a central role in maintaining the health of their eukaryotic hosts and in the cycling of elements like carbon and nitrogen. However, despite their crucial importance, the mechanisms that influence the functioning of microbial communities and their relationship with environmental perturbations are obscure. The study of microbial communities was boosted by tremendous advances in sequencing technologies, and in particular by the possibility to determine genomic sequences of bacteria directly from environmental samples. Indeed, with the advent of metagenomics, it has become possible to investigate, on a previously unparalleled scale, the taxonomical composition and the functional genetic elements present in a specific community. Notwithstanding, the metagenomic approach *per se* suffers some limitations, among which the impossibility of modeling molecular-level (e.g., metabolic) interactions occurring between community members, as well as their effects on the overall stability of the entire system. The family of constraint-based methods, such as flux balance analysis, has been fruitfully used to translate genome sequences in predictive, genome-scale modeling platforms. Although these techniques have been initially developed for analyzing single, well-known model organisms, their recent improvements allowed engaging in multi-organism *in silico* analyses characterized by a considerable predictive capability. In the face of these advances, here we focus on providing an overview of the possibilities and challenges related to the modeling of metabolic interactions within a bacterial community, discussing the feasibility and the perspectives of this kind of analysis in the (near) future.

## Metabolic-Based Interactions and the Microbiome

The advent of high-throughput sequencing platforms (NGS) represents one of the most significant milestones in the field of microbial ecology. The possibility of determining genomic sequences directly from environmental samples, circumventing the culturability issues related to most of the bacterial species, allows to investigate the composition of the microbial communities from taxonomical (microbiome) and functional (metagenome) point of view. Metagenomics, in particular, can be used to identify the metabolic potential of a microbial community in terms of the presence of genes encoding enzymes involved in specific metabolic pathways.

Metabolic interactions are pivotal for maintaining the community processes functions and for structuring the ecology of the host-microbiome unit (Harcombe et al., 2014; Ponomarova and Patil, 2015; Zelezniak et al., 2015). For example, in a community of oceanic plankton the exchange of 2,3-dihydroxypropane-1-sulfonate from the diatom *Thalassiosira pseudonana* to a bacterium from the *Roseobacter* clade has been demonstrated (Durham et al., 2015). Concerning the host-associated microbiota, whose implications for human health and development are well established (Lupton, 2004; Sonnenburg et al., 2005; Ley et al., 2006; Frank et al., 2007; Candela et al., 2008; Turnbaugh et al., 2008; Fukuda et al., 2011; Kozyrskyj et al., 2011; Olszak et al., 2012; Yatsunenko et al., 2012), its composition is the result of complex (and poorly understood) interactions which often depends on metabolic effectors occurring at the molecular level between host and microbes, deriving from long-term co-adaptation and short-term changes of environmental conditions (as exemplified in the hologenome theory; Theis et al., 2016) In the human gut microbiota, one of the most illuminating examples is fucose, a sugar commonly found as glycan component in epithelial cells of the mammal intestine (Terahara et al., 2011). Glycan fucosylation, induced by the presence of specific symbionts (such as *Bacteroides thetaiotaomicron*; Bry et al., 1996), has a role in the foraging of commensal bacteria and, consequently, in the stability of gut microbiota. In turn, a fucose-exposed microbiota improves its host health through different mechanisms, such as the production of short chain fatty acids and the inhibition of pathogen colonization (Pham et al., 2014).

## Toward Predictive Models in Microbial Ecology

Microbial communities can be considered “complex adaptive systems” (Song et al., 2014), where individuals and populations interact, giving rise to system’s higher-order (emergent) properties. Communities are in fact comprised of a network of spatially distributed agents (cells) that respond concurrently to the actions of others (cells). Thus, the behavior of the system (the community) can arise from a variety of interactions (e.g., mutualism, antagonism, parasitism, etc.) between agents and their local environment. In fact, sociomicrobiology is moving from the analysis of single model systems (e.g., *Dictyostelium discoideum*, *Myxococcus xanthus*, *Pseudomonas aeruginosa*; West et al., 2006) to more complex models, as those related to host–microbe interaction and to microbial consortia (Wyatt et al., 2016). In recent years, the application of conceptual frameworks from market economy theory has become popular, trying to predict the evolution of a microbial community (including the cross-talk of their members) over time (Werner et al., 2014; Tasoff et al., 2015). At the same time, various approaches for mathematical modeling of microbial communities have been applied, including Lotka–Volterra models, evolutionary game models, thermodynamically based models, non-linear regression models, trait-based modeling and stoichiometric modeling (reviewed in Song et al., 2014). Tools for the simulation of microbial community behavior have also been developed (Lardon et al., 2011) which may include interaction between host and the microbiota (as the eGUT http://www.biosciences-labs.bham.ac.uk/kreftlab/eGUT.html) or “simpler” environments resembling a Petri-dish context (Harcombe et al., 2014). In general, modeling of communities may rely on top-down or bottom-up approaches, defined as population-level models (PLMs) and individual-based models (IBMs), whereas PLMs are best applied to homogeneous environments and IBMs are mostly useful when heterogeneous environments are considered (Hellweger et al., 2016). The possible outcomes of a predictive microbial ecology model are many, from biomedicine, environment science, and biotechnology (i.e., metabolic engineering), paving the way for “synthetic ecology” (Zomorrodi and Segre, 2016). In this sense, artificial microbial communities can be designed, being geared toward precise and efficient bio-performances and, at the same time, maintaining the resilience and the complexity of “near” native microbial communities. Under this view, practices such as bacteriotherapy (Cammarota et al., 2014) and extra-terrestrial life support projects (Hendrickx et al., 2006) could be tightly and efficiently programmed.

However, these mechanistic models are mostly based on sharp functional definitions of microbial groups (e.g., glucose utilizers, cellulolytic, methanogens, etc.) that are often difficult to reconcile with detailed microbiological and metagenomic data. Indeed, (i) the very same microbial strain can have many different functional abilities (even contrasting, e.g., nitrogen fixation and denitrification in rhizobia; Delgado et al., 2007), (ii) the same functions may be carried out by phylogenetically distant organisms (e.g., nitrogen fixation), and (iii) the microbial strains of a given species can harbor different metabolic abilities (due to the dispensable genome fraction; Medini et al., 2005). Moreover, other important challenges include the identification of (molecular) interactions of populations as well as the quantification of fluxes of nutrients and energy among individuals and populations (Hanemaaijer et al., 2015). It is then crucial to have accurate descriptions (or predictions) of the metabolic phenotypes expressed by either a given microbial strain or groups of organisms present in the microbiome.

## Accurate Phenotypic Predictions with Constraint-Based Metabolic Modeling

The presence of curated repositories integrating biochemical and genetic knowledge (Kegg, Biocyc; Kanehisa and Goto, 2000; Caspi et al., 2016), together with the increased performances of modern genome annotation tools allows reconstructing the metabolic network of an organism from genome sequence data and literature information. More specifically, Genome-scale Metabolic network Reconstructions (GEMREs) integrate, by means of a mathematical formal representation, the set of metabolic reactions occurring in the cell, including information concerning metabolites, biochemical constraints and metabolic enzymes encoding genes (Fondi and Liò, 2015a). This is done by drafting (i) the list of the biochemical reactions that the organism can carry out (resumed from genome annotation and literature information) together with the constraints of those reaction (e.g., reaction reversibility), (ii) an organism-specific biomass assembly reaction, based on the relative abundancies of biomass constituents, and (iii) inputs and outputs (exchanged fluxes) from and to the external environment. A reconstruction, including all these information, can be exported in a computable format (such as JSON or SBML) and queried with different constraint-based (CB) methods to obtain quantitative predictions of growth phenotypes.

The most commonly used CB technique is flux balance analysis (FBA) (Orth et al., 2010), which relies on modeling the biochemical system under investigation with a stoichiometric matrix and a flux vector. This is a compact representation of the reactions as a linear system of differential equations, reporting the association between metabolites and reactions together with the corresponding stoichiometric coefficients. Under FBA a pseudo-steady state condition is assumed, to let the net sum of production and consumption rates of internal metabolites be 0. Under this assumption, it is possible to identify a feasible flux of metabolites optimizing a given objective function (e.g., biomass production). The predictions obtained with this approach can, in turn, be used to design targeted experiments and gain insights into the role of genes in different conditions. Moreover, experimental results (such as growth phenotypes, differential expression data, and metabolic profiles) can be easily integrated into the model with well-defined protocols to perform accurate condition- or tissue-specific simulations.

Given the relative simplicity of this kind of analysis and the close relationship with the biology of model organisms, this approach (with slight variations) has been widely used in bioengineering, physiology, and genome-scale synthetic biology (Hjersted et al., 2007; Feist and Palsson, 2008). For example, the yields of economically important cofactors can be predicted in different conditions (Varma and Palsson, 1993), alternative optimal flux distributions can be identified by means of Flux Variability Analysis (Mahadevan and Schilling, 2003), as well as for prediction of pathogenicity (Bosi et al., 2016) and metabolic rewiring in relation to an environmental adaptation (Fondi et al., 2016). Considering the results that can be obtained with such techniques, as the genome sequencing and biochemical characterization of whole microbial communities becomes increasingly more feasible, the application of CB methods to microbial communities is turning out as a very promising field.

## Metabolic Modeling of Microbial Communities

In the last years, a number of works describing diverse aspects of multi-organism metabolic modeling has testified the growing interest in this field (Biggs et al., 2015; Heinken and Thiele, 2015). Despite the approximations made when analyzing single organisms become more relevant for community modeling, the possibility of integrating meta-omics data (i.e., metagenomics, transcriptomics, proteomics, metabolomics, and fluxomics) on a highly predictive, systems-based framework allowed gaining important insights into basic aspects of microbial ecology (Fondi and Liò, 2015b). These include the prediction of competition/cooperation patterns (Freilich et al., 2011; Chiu et al., 2014), the characterization of symbiotic interactions (Heinken et al., 2013; Shoaie et al., 2013) and the emergence of community response following nutrient modulations (Zhuang et al., 2011). More practical applications include the prediction of probiotics contrasting *Clostridium difficile* infections (Steinway et al., 2015), insights into pathogenesis mechanisms (Bordbar et al., 2010) and the metabolic engineering of consortia to achieve optimality in bioremediation or synthetic biology (Brenner et al., 2008; Brune and Bayer, 2012).

A defining feature of community modeling is the sharp increase in complexity with respect to single-organism CB analyses. In other words, the simplistic assumptions at the basis of FBA (i.e., steady-state, biomass production as objective function) become challenging when applied to model multi-organism metabolic interactions. This made necessary the development of innovative approaches, which are briefly described in **Table 1**. Overall, these methods differ in the scope and complexity of the analyzed community. For instance, dynamics methods based on dFBA are highly predictive for time-resolved analyses, but require a number of parameters which effectively limit their application to small (two or three organisms), well-characterized systems. On the other hand, the enzyme-soup approach relies on simplistic assumptions and limited *a priori* knowledge of the system under study, making it suited for analyzing complex microbial communities (such as the gut microbiota). Overall, current approaches for community metabolic modeling can be divided into: (i) *quantitative methods*, having a high predictive potential but being limited to simple systems due to parameterization and/or *a priori* knowledge required and (ii) *large-scale methods*, providing mostly qualitative insights but applicable to complex microbial communities.

**Table 1:** Overview of the different approaches adopted to perform metabolic modeling of microbial communities.

Approach	Description	References
Compartmentalization	A logical extension of the multiple compartments for organelles in eukaryotic reconstructions. This approach combines multiple GEMREs in a single large stoichiometric matrix, defining a compartment for each organism and transport reactions for the shared metabolites. The objective function used in this case is a linear combination of the individual biomass functions.	Stolyar et al., 2007; Bordbar et al., 2010, Klitgord and Segre, 2010; Shoaie et al., 2013
Community objectives	This strategy, which is implemented in the OptCom tool, extends the Compartmentalization approach introducing an objective function designed at the community level. This allows to effectively model trophic interactions (e.g., commensalism, parasitism, mutualism, etc.) between members of the community, via a series of nested, bi-level optimizations.	Zomorrodi and Maranas, 2012; Shoaie et al., 2013, El-Semman et al., 2014
Dynamic analysis	Instead of using FBA (whose central assumption is the steady state condition), this dynamic approach relies on dFBA, which allows compounds being accumulated or depleted. Instead of producing static “snapshot” of the metabolic states, the dFBA framework provides a dynamic description of the adaptation to changing conditions and nutrients availability. To cope with this totally different framework, a modified version of OptCom has been tailored to carry out dynamic analyses (dOptCom). Despite the interesting results obtained with this approach, the application of dFBA is severely hindered by two factors: (i) it is computationally demanding and (ii) it requires some kinetic parameters (e.g., for growth-limiting metabolites). A major consequence is the reduced scale of the system that can be analyzed with this approach, with respect to other methods.	Tzamali et al., 2011; Zhuang et al., 2011, Hanly et al., 2012; Chiu et al., 2014, Hanly and Henson, 2014; Harcombe et al., 2014
Spatially resolved	This approach introduces the study of bacterial spatial diffusion and the resulting structure of (simple) microbial communities. COMETS, for example, uses dynamic flux balance analysis (dFBA) to perform time-dependent metabolic simulations of microbial ecosystems, bridging the gap between stoichiometric and environmental modeling.	Gorochowski et al., 2012; Harcombe et al., 2014, Phalak et al., 2016
Enzyme soup	Radically different from the other methods, the enzyme-soup approach completely neglects any inter-organism boundary concept. Reactions are not assigned to different species, as the whole community is treated as a “soup” of enzymes. Since a number of biomass components are shared in the community, the biomass function has a generalized formulation, representing the biomass of the whole community. In accordance with its premises, this approach focuses on depicting the metabolic potential of microbial communities, bypassing the problem of inter-organism interactions. Due to the simple nature of its assumptions, this method can be easily applied to large complex communities, given the experimental support of meta-omic data.	Taffs et al., 2009; Tobalina et al., 2015
Graph-based	Methods defined as graph-based have been used to identify competition or cooperation patterns between bacteria. According to this framework, the stoichiometric matrix is used to generate graph connecting metabolites, with edges directed from substrates to products. Nodes with in-degree/out-degree ratio equal to 0 represent metabolites (*seeds*) which are consumed but not produced, and therefore must be supplied to the network. The assessment of seed sets for multiple organisms allows to evaluate the metabolic basis of competition/cooperation. Since inferences are made regardless of stoichiometry and flux analysis, this approach shows a remarkable robustness when applied to poor-quality reconstructions, which might affect conclusions made using FBA-based methods.	Borenstein et al., 2008; Levy and Borenstein, 2013
Network expansion	This method encompasses an agglomerative algorithm (Network Expansion), which iteratively add reactions to an initial set of reactions/metabolites, aiming at identifying emergent properties of the growing metabolic network. The algorithm has been adapted to suit the case of microbial community analysis, studying the properties of pairwise combinations of bacteria. Basically, starting from an initial set of reactions from both the microbes, this method iteratively expands the network with a pool of reactions from both organisms, under the assumptions that metabolic intermediates can be shared. The application of this method allowed to identify emergent biosynthetic capacities for a large number of bacterial pairs.	Handorf et al., 2005; Christian et al., 2007, Steinway et al., 2015

This simple distinction highlights one current limitation of metabolic modeling methods, that is, the lack of quantitative methods easily scalable to large-scale communities. Although the presence of experimental data (such as meta-omics) can be exploited to improve the biological significance of the predictions obtained in face of the increasing complexity, the development of novel innovative methods overcoming the current limitations is indeed a priority. This includes also (i) the combination of different approaches to obtain hybrid methods optimizing the trade-off between quantitative predictions and scale of the systems and, (ii) the development of integrative frameworks to better combine meta-omics data with metabolic reconstructions. An example of the latter is the dynamic modeling of gut microbiota composition to identify bacteria inhibiting *C. difficile*, performed integrating longitudinal metagenomics data with the network expansion method (Steinway et al., 2015).

Another technical challenge limiting the application of CB methods to complex communities is the quality of GEMREs that can be used. In fact, prediction of metabolic fluxes maximizing a defined objective function requires high-quality metabolic reconstructions (generated with precise protocols; Thiele and Palsson, 2010) to achieve consistency with the actual biology of the organisms accounted by the reconstruction. However, the model typically requires further refinement (such as integration of literature and/or extant physiological data to identify potential gaps) and validation steps, which can be quite time (and resource) consuming. Resultantly, the protocol used to obtain GEMREs of single organisms cannot be extended to large datasets due to the long time required to carry out these analyses and/or potential knowledge gaps for some organisms (such as unculturable bacteria) hindering the application of bottom-up reconstruction approaches.

General strategies have been developed to rapidly obtain GEMREs for many organisms. These are based on automatic reconstruction from genomes (or binned metagenomic contigs), or comparative approaches relying on orthologous genes with “reference” organisms for which high-quality GEMREs are available. Either way, the obtained draft-quality GEMREs require additional refinement steps to fill potential reaction gaps. Perhaps the most notable example of such large-scale analysis is the metabolic reconstruction of 773 human gut microbes using a semi-automatic comparative metabolic reconstruction method (Magnusdottir et al., 2017). Although the analysis of these GEMREs revealed good consistency with known functional features of gut microbiota (e.g., carbon source compounds degradation; Flint et al., 2012), the authors specified the infeasibility of this approach to recover (accurate) quantitative predictions, due to the absence of species/condition-specific information (i.e., the breakdown of biomass components). On the other hand, qualitative insights such as prediction of growth-supporting media seem to be less affected by this kind of approximations (Feist and Palsson, 2010).

Altogether, this points to the need of (i) established protocols (such as Thiele and Palsson, 2010) to develop and curate GEMREs for large-scale datasets and (ii) public resources to facilitate this task (see Magnusdottir et al., 2017). In particular, we specifically stress the lack of a data repository describing the biomass composition of different organisms in a variety of conditions. Indeed, such knowledge could be easily integrated in existing reconstruction pipelines and would allow obtaining more biologically relevant GEMREs.

## Strengths and Weaknesses of Community Models

**FIGURE 1:**
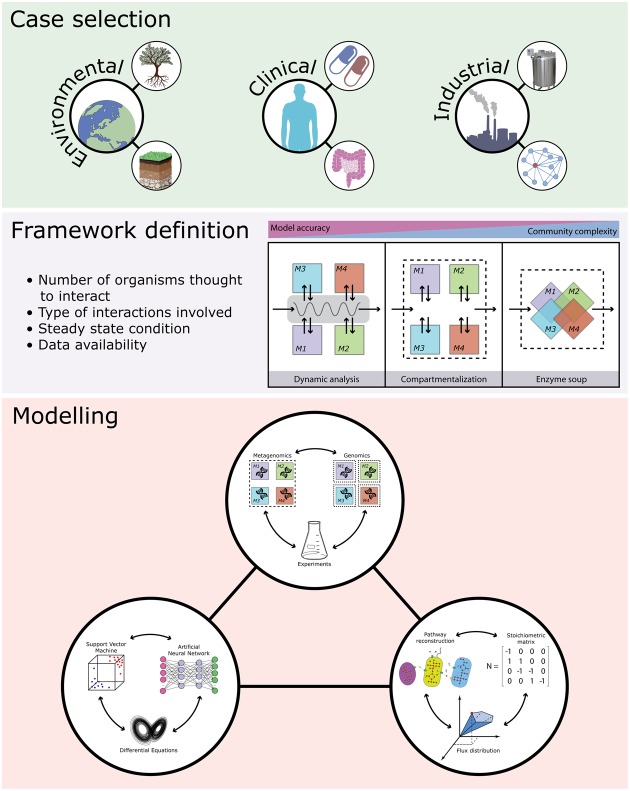
Overview of the main steps and applications in bacterial community metabolic modeling. Microbiomes with environmental, clinical, and industrial relevance (top panel) are selected and models are chosen based on a balance between the desidered model accuracy and the complexity of the microbiome (central panel). Then modeling is applied and combined with information from metagenomic data and genome sequences (and phenotypic/biochemical information) from cultivated microorganisms (lower panel).

Knowledge-driven metabolic engineering of bacterial communities is an emerging field which might shed light on some of the most puzzling biological questions regarding clinical problems (e.g., drug–bacteria interactions; Ye et al., 2014), industrial production design (e.g., enhancing secondary metabolites production; Kim et al., 2016), and environmental safety/health (e.g., bioremediation; Rein et al., 2016) (**Figure 1**). Several efforts have been directed at characterizing the interactions between bacterial pathogens and their host, aiming at designing probiotic formulations to recover damaged communities (such as the human gut microbiota following *C. difficile* infection; Buffie et al., 2015), or able to directly suppress pathogen proliferation (Buffie et al., 2015). The metabolic repertoire shared by complex bacterial communities, such as those living in the human gut, has been explored using semi-automated approaches to reconstruct a large set of metabolic models intertwining genomic, metagenomic, and metabolic information (Magnusdottir et al., 2017). Microbial consortia can, in principle, perform complex reactions requiring multiple steps that can be cell- or community-specific (Brenner et al., 2008). Understanding the communication systems underpinning bacterial communities represents a crucial step for the rational design of microbial consortia able to maximize the production of different compounds or for the production of hybrid communities, composed of natural and engineered bacteria, to be used in bioremediation processes (Brune and Bayer, 2012). Despite all the advances made in the integration of omics data into community-level models, more work is needed to overcome limitations imposed by current computational and experimental procedures.

Rational design of engineered microbial communities can be translated into specific services (e.g., bioremediation, metabolites productions, protection against pathogens, etc.). However, this requires the precise annotation of metabolic functions to the species present in the communities, and this can be not feasible, especially when complete and annotated genomes are missing. Despite the recent progress in genome reconstruction from metagenomes (Nielsen et al., 2014), the gap between omics information acquired and reference genomes assembled and available in public databases is still to be filled. Methods for binning metagenomic sequences into taxonomic groups are mainly based on the different DNA composition (i.e., unsupervised methods combining *k-mer* frequencies with coverage information; Alneberg et al., 2014) or on pairwise comparisons with taxonomic annotated sequences (i.e., supervised methods based on sequence similarity; Brady and Salzberg, 2009; Wood and Salzberg, 2014). Even if these methods can be used for partitioning genomes into different “biological units,” thus allowing metabolic models reconstruction, at present it is not feasible to recover all the genomes that compose an entire natural community and, consequently, perform metabolic modeling of the whole microbial community. Another bottleneck in community level metabolic modeling is the generation of a model for each component of the community. Indeed, microbial consortia are composed by thousands of strains and producing a different, curated and reliable model for each strain would be very demanding in terms of costs and time (**Figure 1**). For this reason, the automatic generation of models from genomic and metagenomics data is a mandatory step to increase the resolution power of the community model, especially in natural environment where, in principle, every single cell takes part in maintaining the homeostasis of a particular niche.

On the other hand, while genomics and metagenomics have provided many insights into the role of bacteria in determining potential functional features of a given environment, they both provide a static snapshot of a community, thus failing to deliver a dynamic and fully functional representation. Consequently, obtaining an accurate dynamic model of a community would require longitudinal metagenomics or, more in general, methods to infer growth dynamics of single bacterial species from metagenomics (Korem et al., 2015). However, this might not be straight-forward, since time-resolved metagenomics shows that some species can grow faster than others increasing their abundance quickly enough to significantly change the whole community structure (Bacci et al., 2015). Therefore, given the paradigm of CB metabolic modeling, this could effectively represent a problem when trying to infer the metabolic phenotype of a microbial community. Similarly, it is unlikely that all the members of a microbial consortium are optimally geared toward biomass production. As a consequence, the steady-state assumption (that is the foundation of FBA analyses) may not hold true during simulations.

All these factors force to discuss about which assumptions made in the context of single-organism metabolic modeling can be still tolerated when trying to accurately infer the (metabolic) dynamic of a bacterial community. Indeed, each framework proposed hitherto takes into account different aspects of microbial interactions leaving to researchers the burden of choosing the kind of model that best fits their needs; this decision should be primarily based on the information available as well as on the resolution level that is possible to achieve (**Figure 1**). As ‘omic sciences become more and more affordable and sensible, their integration into community-level metabolic models is mandatory to achieve a systems level understanding of these biological entities. This highlights the necessity of a working scheme designed to handle large-scale, community-level reconstructions and to derive quantitative insights.

## Author Contributions

All the authors contributed in conceiving, preparing and revising the manuscript. All the authors approved the manuscript and agreed to be accountable for all aspects of the presented work.

## Conflict of Interest Statement

The authors declare that the research was conducted in the absence of any commercial or financial relationships that could be construed as a potential conflict of interest.
